# Analysis of a surface imaging system using a six degree‐of‐freedom couch

**DOI:** 10.1002/acm2.13697

**Published:** 2022-07-12

**Authors:** Xiaodong Zhao, Elizabeth L. Covington, Richard A. Popple

**Affiliations:** ^1^ Department of Radiation Oncology University of Alabama at Birmingham Birmingham Alabama USA

**Keywords:** six degree‐of‐freedom couch, stereotactic radiosurgery, surface‐guided radiotherapy, surface imaging

## Abstract

**Purpose:**

To validate surface imaging (SI)‐reported offsets using a six degree‐of‐freedom couch and an anthropomorphic phantom for commissioning and routine quality assurance of an SI system used for stereotactic radiosurgery (SRS).

**Methods:**

An anthropomorphic phantom with a radiopaque ball bearing (BB) placed either anterior, midline, or posterior, was tracked with SI with a typical SRS region of interest. Couch motion in all six degrees of freedom was programmed and delivered on a linac. SI system logs were synchronized with linac trajectory logs. Ten random couch positions were selected at couch 0°, 45°, 90°, 270°, 315° with megavolt (MV) images taken to account for couch walkout. The SI residual error (*ε*), the difference between SI reported offset and MV or trajectory log position, was calculated. Residual errors were measured with and without one SI pod blocked.

**Results:**

The median [range] of magnitude of translational *ε* was 0.13 [0.07, 0.21], 0.16 [0.11, 0.26], 0.61 [0.50, 0.68], 0.49 [0.42, 0.55], 0.55 [0.38, 0.72] mm for couch rotations of 0°, 45°, 90°, 270°, 315°, respectively, for the midline BB and no pod blocked. The range of all translational *ε* from all couch angles (with and without pod block) at different BB positions is [0.05, 0.96] mm. The absolute range of difference when changing BB position when no pod is blocked in median translational *ε* is [0.01, 0.40] mm with the maximum at BB posterior. The absolute range of difference when not changing BB positions with and without pod block in median translational *ε* is [0.01, 0.37] mm with the maximum at BB posterior and couch 315°.

**Conclusion:**

SI system and linac trajectory log analysis can be used to assess SI system performance with automated couch motion to validate SI accuracy.

## 1 INTRODUCTION

Surface imaging (SI) has been used during frameless stereotactic radiosurgery (SRS) to monitor intrafraction patient motion.^1^, ^2^, ^3^, ^4^, ^5^, ^6^, ^7^, ^8^, ^9^, ^10^, ^11^ Using flattening filter‐free volumetric‐modulated arc therapy with automated motion via Hyperarc (Varian Medical Systems, Palo Alto, CA, USA) enables efficient SRS treatments with one isocenter for multiple brain metastases.^12^ To ensure that there is no significant target movement during treatment, it is very important to commission and evaluate the accuracy of the SI system used due to the small target size with small or zero target margins.

We recently commissioned an IDENTIFY (Varian Medical Systems), an SI system, to monitor intrafraction motion for all intracranial treatments delivered on an Edge (Varian Medical Systems) linear accelerator. Similar to other SI systems, IDENTIFY has three pods, with each pod containing two cameras and one projector. IDENTIFY uses a reference surface, which is created from the treatment planning computed tomography (CT) or captured by the SI system in real time. A region of interest (ROI) is selected for monitoring (e.g., the open face region of a thermoplastic mask), and its position is compared with the reference surface during treatment to monitor the patient's movement.^13^, ^14^ The differences in six degrees of freedom, including longitudinal, lateral, vertical, pitch, roll, and yaw, are displayed for monitoring, and the treatment can be manually stopped when the treatment team finds that offsets are larger than the intrafraction motion thresholds set by the clinic.

The use of IDENTIFY for SRS intrafraction monitoring has not been extensively studied. In previous studies,^15^, ^16^ similar SI systems were tested against kilovoltage (kV), megavoltage (MV), and cone‐beam CT (CBCT) images, where the phantom was stationary after the CBCT setup and the choice of phantom was not similar to the patient setup. In this paper, we present a novel methodology for IDENTIFY commissioning using the six degree‐of‐freedom couch with MV portal imaging verification on an anthropomorphic phantom. This study aims to quantify the accuracy of the IDENTIFY SI system with known couch positions from linac trajectory logs and MV imaging to validate SI‐reported offsets on an anthropomorphic phantom for commissioning and routine quality assurance of an SI system used for SRS.

## 2 MATERIALS AND METHODS

**FIGURE 1:**
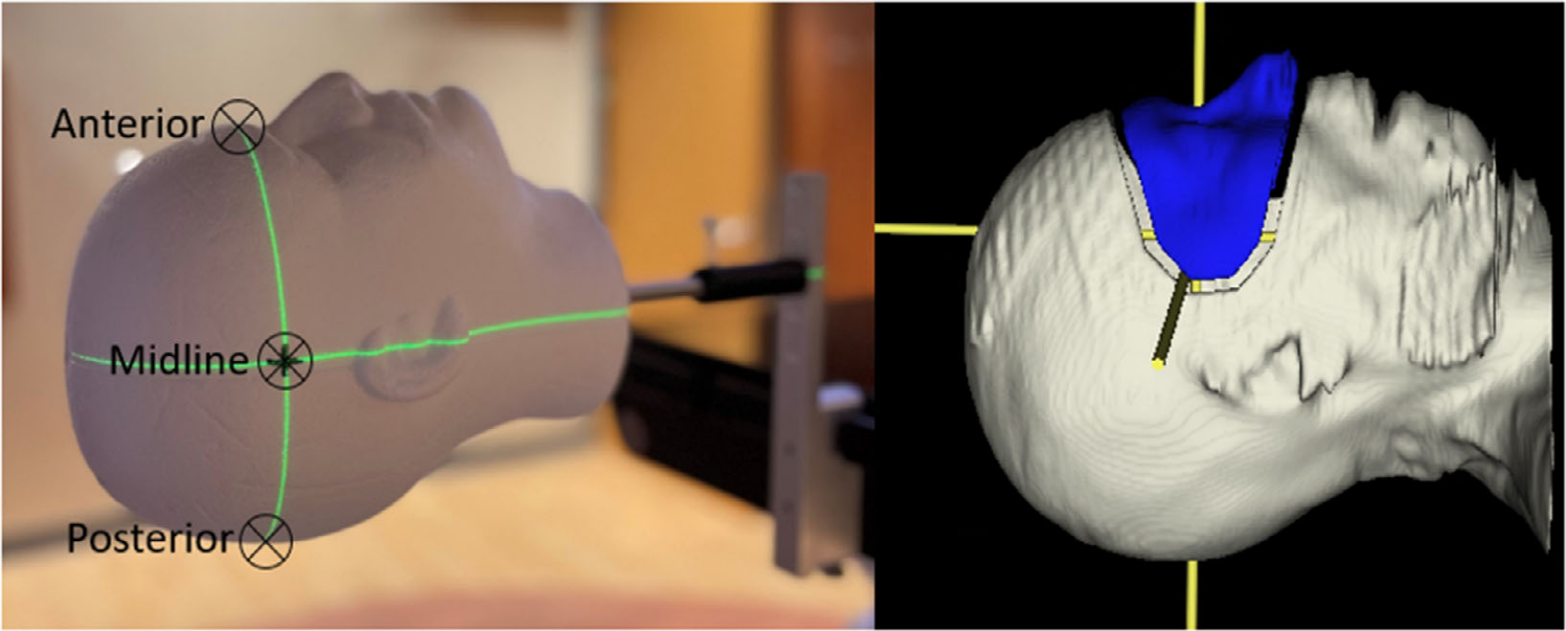
(Left) Setup of anthropomorphic Styrofoam phantom with ball bearing (BB) placed at anterior, midline, and posterior of the phantom, with the same tracking region of interest (ROI) contoured in blue (right)

An anthropomorphic Styrofoam phantom (Floracraft, Ludington, MI, USA) with a radiopaque tungsten carbide ball bearing (BB) was tracked with the SI with an ROI representative of a typical SRS ROI. The BB was placed either anterior (ant), midline (mid), or posterior (pos) to the phantom, as shown in Figure 1. The phantom was aligned by placing the BB at the isocenter with orthogonal kV imaging at couch 0°. Couch motion was programmed in developer mode and delivered on an Edge linac. The.xml file used is attached in Supporting Information S2. Ten random couch positions in a ±1.5 mm and ±0.5° range were selected at couch rotations of 0°, 45°, 90°, 270°, and 315° with MV images taken to account for couch walkout. The 10 couch positions programmed for each couch angle are the same. We programmed the beam to deliver an open field at a gantry angle of 0° and collimator angle of 0° for 50 MU at each couch position. The MV image was acquired halfway at each couch position, that is, when 25 MU was delivered, on an electronic portal imaging device. We also repeated the same experiment with one of the three pods blocked to mimic a common scenario when pods are blocked by the gantry. It is common during fields with nonzero couch angles to have a pod blocked for the majority of the field delivery (e.g., left pod with couch 90°). We repeated the experiment of 10 couch positions for each couch angle with and without pods blocked for the three BB positions.

The IDENTIFY logs were synchronized with the linac trajectory log by minimizing the mean square difference between the translation offset magnitudes. This strategy effectively uses the transitions between positions to synchronize the logs. The IDENTIFY residual error (*ε*), the difference between SI reported offset and MV or trajectory log position, was calculated.^17^ The MV images were used to account for the offset due to couch walkout that is not included in the trajectory logs but is a component of the SI reported offsets. Therefore, we subtracted the couch walkout from the SI reported offsets, and the residual errors were purely SI system uncertainties. For the lateral and longitudinal directions, *ε* was calculated from MV images, considering that it is not possible to subtract couch walkout from linac trajectory logs. A threshold method was used to identify the BB from the MV images and calculate the BB movement at each couch position. When an appropriate threshold is applied, the delineated circle matches the BB's physical diameter. For the vertical direction, couch walkout was determined to be less than 0.05 mm,^18^ so the trajectory log value was used. Linac trajectory logs were used to calculate *ε* in the vertical direction and in all three rotation directions. The residual errors for each axis are given by

(1) $$\begin{array}{ccc} \varepsilon_{\mathrm{Lng}}^{\mathrm{BB},\mathrm{pod}}\left(\theta ,\;i\right) & = & {\mathrm{Lng}}^{{\mathrm{IDENTIFY}}^{\mathrm{BB},\mathrm{pod}}}\\ & & \left(\theta ,\;t_{i}\right)-{\mathrm{Lng}}^{{\mathrm{MV}}^{\mathrm{BB},\mathrm{pod}}}\left(\theta ,\;i\right), \end{array}$$

(2) $$\begin{array}{ccc} \varepsilon_{\mathrm{Lat}}^{\mathrm{BB},\mathrm{pod}}\left(\theta ,\;i\right) & = & {\mathrm{Lat}}^{{\mathrm{IDENTIFY}}^{\mathrm{BB},\mathrm{pod}}}\\ & & \left(\theta ,\;t_{i}\right)-{\mathrm{Lat}}^{{\mathrm{MV}}^{\mathrm{BB},\mathrm{pod}}}\left(\theta ,\;i\right), \end{array}$$

(3) $$\begin{array}{ccc} \varepsilon_{\mathrm{Vrt}}^{\mathrm{BB},\mathrm{pod}}\left(\theta ,\;i\right) & = & {\mathrm{Vrt}}^{{\mathrm{IDENTIFY}}^{\mathrm{BB},\mathrm{pod}}}\\ & & \left(\theta ,\;t_{i}\right)-{\mathrm{Vrt}}^{{\mathrm{LinacLog}}^{\mathrm{BB},\mathrm{pod}}}\left(\theta ,\;t_{i}\right), \end{array}$$

(4) $$\begin{array}{ccc} \varepsilon_{\varphi }^{\mathrm{BB},\mathrm{pod}}\left(\theta ,\;i\right) & = & \varphi^{{\mathrm{IDENTIFY}}^{\mathrm{BB},\mathrm{pod}}}\\ & & \left(\theta ,\;t_{i}\right)-\varphi^{{\mathrm{LinacLog}}^{\mathrm{BB},\mathrm{pod}}}\left(\theta ,\;t_{i}\right), \end{array}$$

where *θ* is the couch angle, i∈[0,10] is the couch position, $t_{i}$ is the timestamp of the *i*th couch position, *φ* indicates the rotation direction (roll, pitch, or yaw), BB indicates the BB position, and pod indicates which pod is blocked. For all data, the reference position corresponding to no offsets (*ε* = 0) is at couch angle 0° with no pods blocked (i.e., *i *= 0, *θ *= 0, pod = no). The magnitude of the residual translation error is given by

(5) $$\begin{array}{ccc} & & \left|\varepsilon^{\mathrm{BB},\mathrm{pod}}\left(\theta ,\;i\right)\right|\\ & & =\sqrt{\varepsilon_{\mathrm{Lng}}^{\mathrm{BB},\mathrm{pod}}{\left(\theta ,\;i\right)}^{2}+\varepsilon_{\mathrm{Lat}}^{\mathrm{BB},\mathrm{pod}}{\left(\theta ,\;i\right)}^{2}+\varepsilon_{\mathrm{Vrt}}^{\mathrm{BB},\mathrm{pod}}{\left(\theta ,\;i\right)}^{2}}. \end{array}$$

The mean and standard deviations of each six degree‐of‐freedom component are compared across all BB positions, pod blockage, and couch rotations. The magnitude of the residual translation error is compared in each scenario as well. Because the magnitude of the residual error was not distributed normally, we reported the median, range, and Wilcoxon rank‐sum test *p*‐value. The median of the residual translation error at no pod block with BB at anterior or posterior was compared to the median of the residual translation error at no pod block with BB at midline with the Wilcoxon rank‐sum test. The median of the residual translation error at each BB position with the left or right pod block was compared to the median of the residual translation error at the same BB position with no pod block with the Wilcoxon rank‐sum test. For example, the median of magnitude of the residual translation error for BB midline, left pod block, at couch angle 45° is expressed as $\mathrm{median}|\varepsilon^{\mathrm{mid},\mathrm{lt}}\left(45^{\circ }\right)|$, while the mean of roll of the residual error for BB midline, no camera block, at couch angle 0° is expressed as $\mathrm{mean}\;\varepsilon_{\mathrm{Roll}}^{\mathrm{mid},\mathrm{no}}\left(0^{\circ }\right)$.

## 3 RESULTS

**FIGURE 2:**
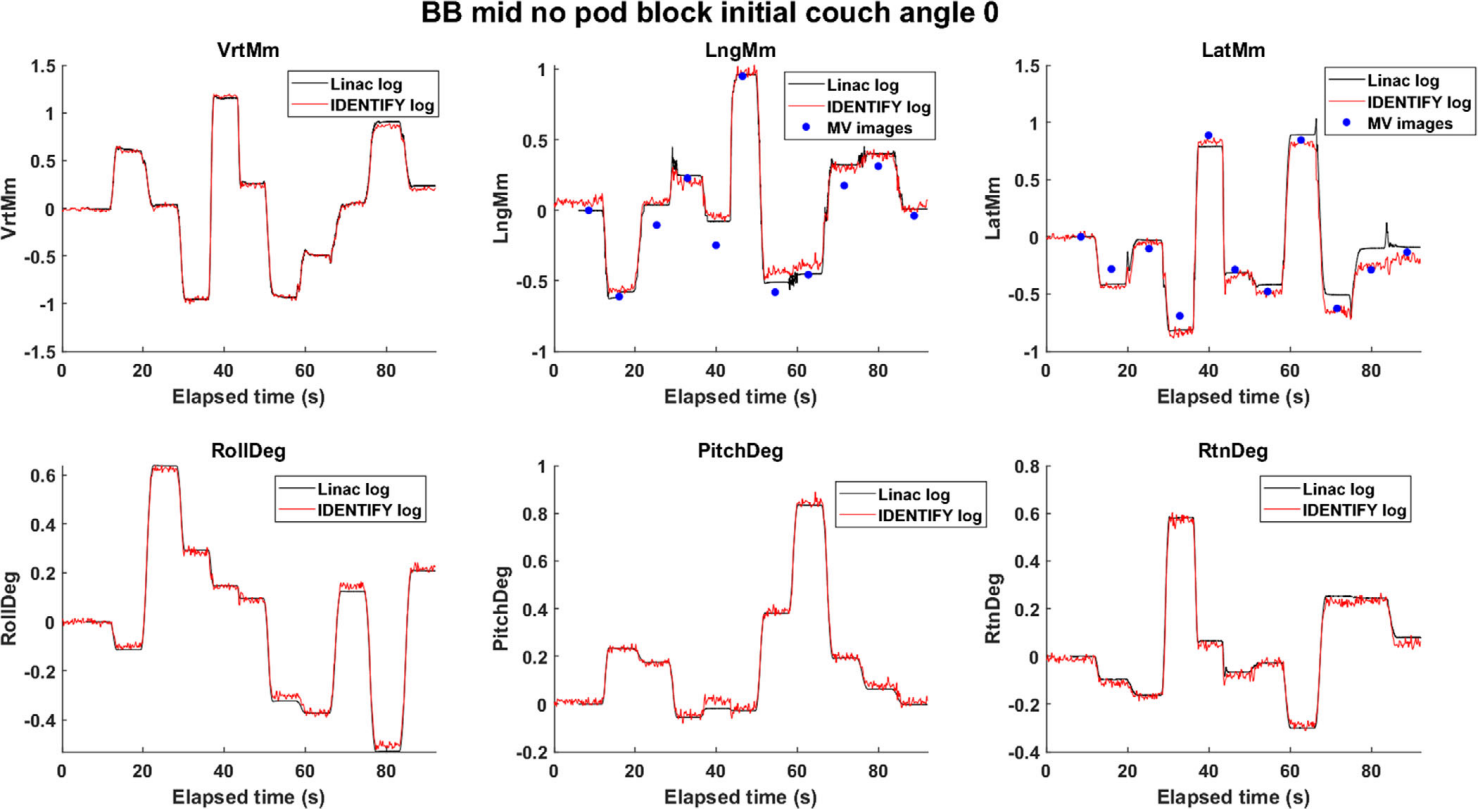
Comparison of phantom position reported by linear accelerator trajectory logs, surface imaging logs, and megavolt (MV) image analysis at couch 0° with midline ball bearing (BB) position and no pod block. When the couch angle, BB position or pod blocking were different, a similar pattern was observed. Here, the couch moved to 10 preprogrammed randomly chosen locations within a range of ±1.5 mm and ±0.5°. The same 10 preprogrammed couch positions were repeated for different couch angle, BB position or pod blocking scenarios. There are 11 blue dots indicated by MV images because the first position is always neutral for any couch angle

**FIGURE 3:**
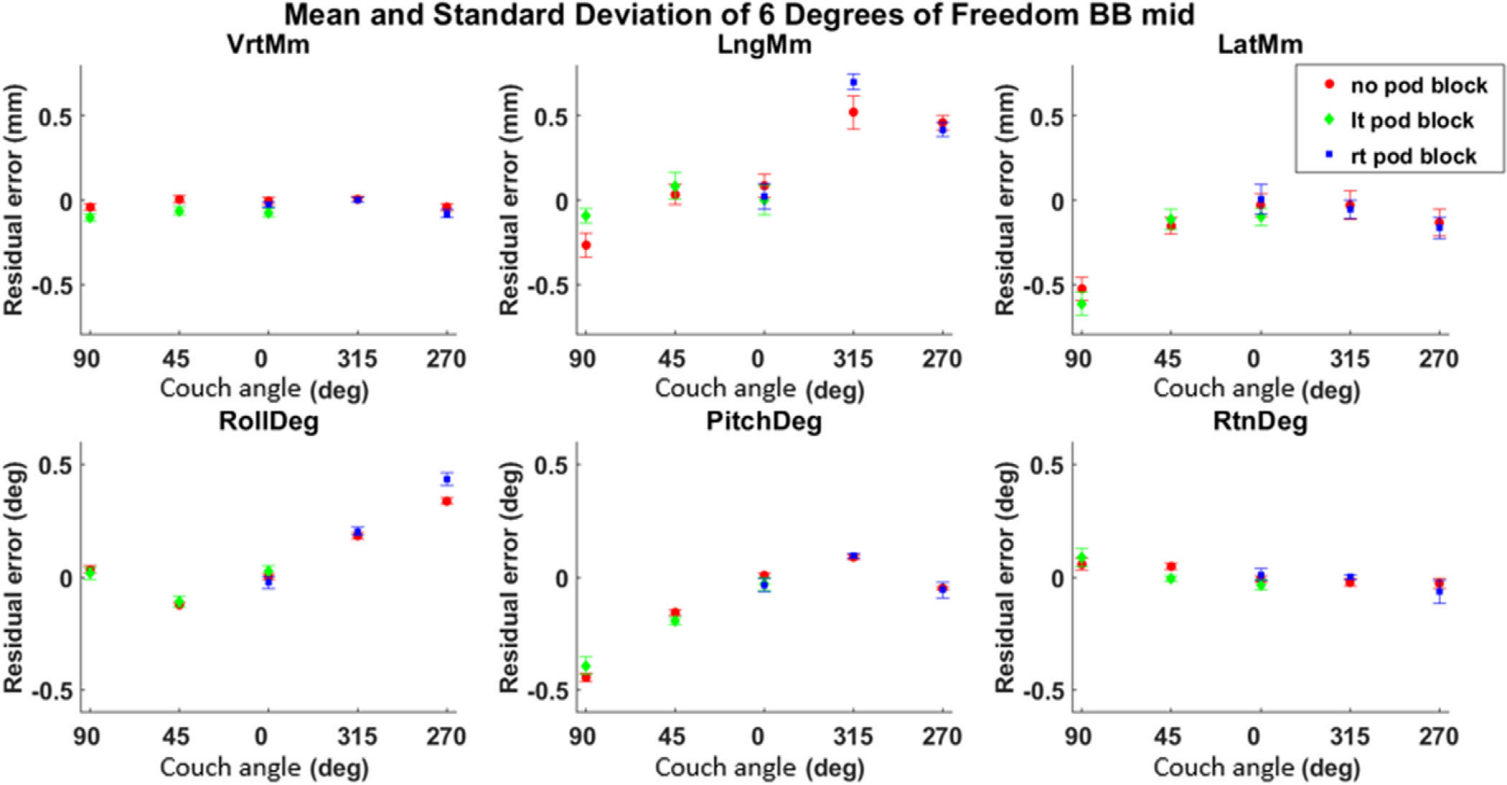
Surface imaging (SI) residual error for no pod block mean (red dot) and standard deviation (red error bar) of vertical, longitudinal, lateral directions in mm, roll, pitch and rotation in degree is shown for couch angles 90°, 45°, 0°, 315°, 270°. Left pod block data are shown in green diamonds, and right pod block data are shown in blue squares. Longitudinal and lateral residual errors were analyzed from IDENTIFY logs and megavolt (MV) imaging data, whereas the other four degrees were from IDENTIFY logs and linac trajectory logs

Figure 2 shows a comparison of an IDENTIFY log, linac trajectory log, and MV images detected position for couch angle 0°, BB position midline, and no pod block as an example. In Figure 3, the mean and standard deviation of the residual error for all six degrees of freedom are plotted against the couch angle for the BB position midline with and without pod blocking. For the other two BB positions, shown in Table S1, pod block and no pod block data showed similar results. The vertical direction showed negligible residual error, and the longitudinal and lateral residual errors were less than 0.50 mm in magnitude. Table rotation (yaw) showed negligible residual error, and pitch and roll residual errors were less than 0.50° in magnitude. With a pod block, the largest difference between residual error was 0.41 mm.

Table 1 shows the median and range of translational error $|\varepsilon^{\mathrm{mid},\mathrm{no}}\left(\theta \right)|$ for all five couch angles at the BB position midline and no pod block. For all couch angles, the range of median $|\varepsilon^{\mathrm{mid},\mathrm{no}}\left(\theta \right)|$ is [0.13, 0.61] mm. Table 1 is used as the baseline to calculate the difference of different BB positions in Table 2. Table 2 shows the difference between the median and Wilcoxon rank‐sum test *p*‐values at $|\varepsilon^{\mathrm{BB},\mathrm{no}}\left(\theta \right)|$ to $|\varepsilon^{\mathrm{mid},\mathrm{no}}\left(\theta \right)|$, where BB=antorpos. The biggest difference of median is from the couch 90° BB position posterior, which is ‐0.40 mm. Table 3 shows the difference between the median and Wilcoxon rank‐sum test *p*‐values at $\varepsilon^{\mathrm{BB},\mathrm{pod}}\left(\theta \right)\;$ to $\varepsilon^{\mathrm{BB},\mathrm{no}}\left(\theta \right)$, where BB=ant,mid,orpos and pod=lforrt. For each pod block scenario, it is compared to the same BB position, that is, for example, $\varepsilon^{\mathrm{ant},\mathrm{lt}}\left(\theta \right)\;$ to $\varepsilon^{\mathrm{ant},\mathrm{no}}\left(\theta \right)$.

**TABLE 1:** Summary of median and range of translational error $|\varepsilon^{\mathrm{mid},\mathrm{no}}\left(\theta \right)|$, mean and standard deviation of $\varepsilon_{\mathrm{Roll}}^{\mathrm{mid},\;\mathrm{no}}\left(\theta \right)$, $\varepsilon_{\mathrm{Pitch}}^{\mathrm{mid},\;\mathrm{no}}\left(\theta \right),$ and $\varepsilon_{\mathrm{Yaw}}^{\mathrm{mid},\;\mathrm{no}}\left(\theta \right)$ at ball bearing (BB) position midline and no pod block, which is used as the baseline for Tables 2 and 3

Table angle, *θ* (°)	Median $|\varepsilon^{\mathrm{mid},\mathrm{no}}\left(\theta \right)|$ (mm)	Min $|\varepsilon^{\mathrm{mid},\mathrm{no}}\left(\theta \right)|$ (mm)	Max $|\varepsilon^{\mathrm{mid},\mathrm{no}}\left(\theta \right)|$ (mm)	Mean $\varepsilon_{\mathrm{Roll}}^{\mathrm{mid},\;\mathrm{no}}\left(\theta \right)$ (°)	Std $\varepsilon_{\mathrm{Roll}}^{\mathrm{mid},\;\mathrm{no}}\left(\theta \right)$ (°)	Mean $\varepsilon_{\mathrm{Pitch}}^{\mathrm{mid},\;\mathrm{no}}\left(\theta \right)$ (°)	Std $\varepsilon_{\mathrm{Pitch}}^{\mathrm{mid},\;\mathrm{no}}\left(\theta \right)$ (°)	Mean $\varepsilon_{\mathrm{Yaw}}^{\mathrm{mid},\;\mathrm{no}}\left(\theta \right)$ (°)	Std $\varepsilon_{\mathrm{Yaw}}^{\mathrm{mid},\;\mathrm{no}}\left(\theta \right)$ (°)
0	0.13	0.07	0.21	0.00	0.01	0.01	0.01	‐0.01	0.01
45	0.16	0.11	0.26	‐0.12	0.01	‐0.16	0.01	0.05	0.01
90	0.61	0.50	0.68	0.03	0.02	‐0.44	0.02	0.06	0.03
270	0.49	0.42	0.55	0.34	0.01	‐0.05	0.01	‐0.03	0.02
315	0.55	0.38	0.72	0.18	0.01	0.09	0.01	‐0.02	0.02

**TABLE 2:** Summary of medians of translational error, at ball bearing (BB) position anterior, midline, and posterior with no pod block, *p*‐values are compared to BB position midline

BB position	BB midline	BB anterior		BB posterior	
Table angle, *θ* (°)	Median $|\varepsilon^{\mathrm{mid},\mathrm{no}}\left(\theta \right)|$ (mm)	Median $|\varepsilon^{\mathrm{ant},\mathrm{no}}\left(\theta \right)|$ (mm)	*p*‐Value	Median $|\varepsilon^{\mathrm{pos},\mathrm{no}}\left(\theta \right)|$ (mm)	*p*‐Value
0	0.13	0.14	0.24	0.11	0.74
45	0.16	0.23	0.00	0.19	0.36
90	0.61	0.40	0.00	0.20	0.00
270	0.49	0.35	0.00	0.21	0.00
315	0.55	0.37	0.00	0.41	0.01

**TABLE 3:** Summary of medians of translational error at the ball bearing (BB) position anterior, midline, and posterior with different pod block scenarios, *p*‐values are compared to their same BB position and no pod block counterpart

BB position		BB anterior			BB midline			BB posterior		
Pod block	Table angle, *θ* (°)	No pod block median $|\varepsilon^{\mathrm{ant},\mathrm{no}}\left(\theta \right)|$ (mm)	Pod block median $|\varepsilon^{\mathrm{ant},\mathrm{pod}}\left(\theta \right)|$ (mm)	*p*‐Value	No pod block median $|\varepsilon^{\mathrm{mid},\mathrm{no}}\left(\theta \right)|$ (mm)	Pod block median $|\varepsilon^{\mathrm{mid},\mathrm{pod}}\left(\theta \right)|$ (mm)	*p*‐Value	No pod block median $|\varepsilon^{\mathrm{pos},\mathrm{no}}\left(\theta \right)|$ (mm)	Pod block median $|\varepsilon^{\mathrm{pos},\mathrm{pod}}\left(\theta \right)|$ (mm)	*p*‐Value
Left pod block	0	0.14	0.25	0.01	0.13	0.14	0.12	0.11	0.23	0.00
	45	0.23	0.42	0.00	0.16	0.19	0.95	0.19	0.32	0.00
	90	0.40	0.63	0.00	0.61	0.64	0.21	0.20	0.38	0.00
Right pod block	0	0.14	0.13	0.65	0.13	0.09	0.51	0.11	0.24	0.00
	270	0.35	0.33	0.90	0.49	0.48	0.26	0.21	0.23	0.74
	315	0.37	0.50	0.04	0.55	0.70	0.00	0.41	0.79	0.00

**FIGURE 4:**
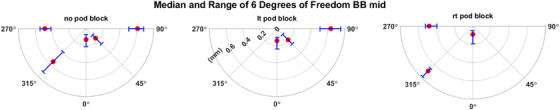
Median (red dots) and range (blue error bar) of magnitude of translation residual error $|\varepsilon^{\mathrm{mid},\mathrm{pod}}\left(\theta \right)|\;$ (mm), from all 50 random couch positions from five couch angles at ball bearing (BB) position midline with no pod block (left), with left pod block (middle), and with right pod block (right). The scale of the plots is shown in the middle figure

**FIGURE 5:**
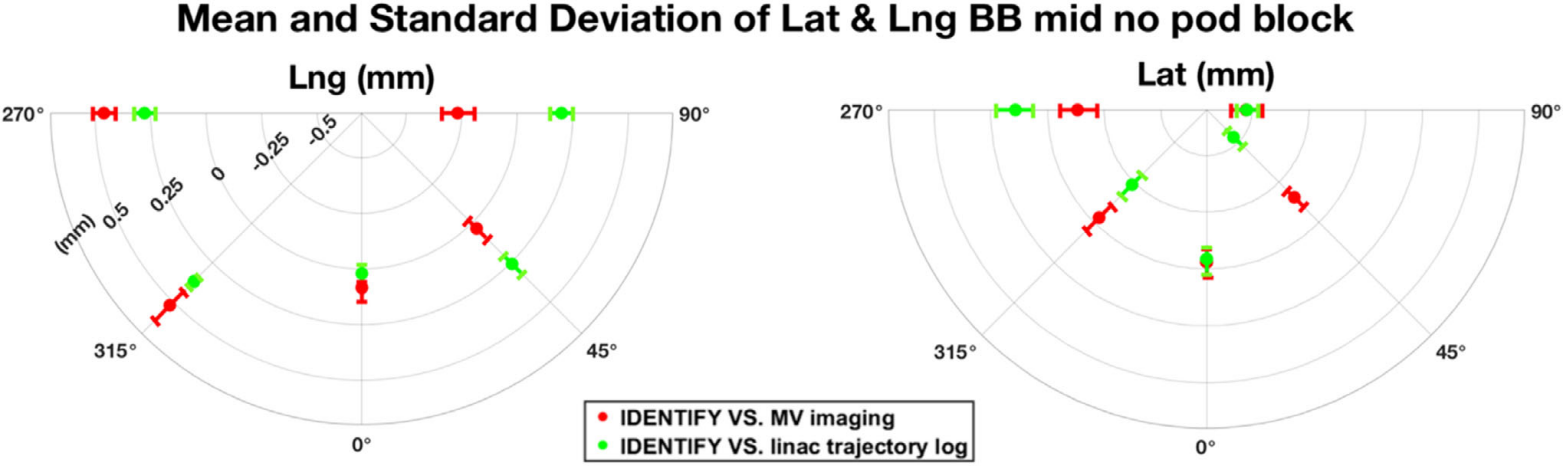
Residual error of longitudinal direction (left) and lateral direction (right) in mm of IDENTIFY compared to linac trajectory log (green) and megavolt (MV) imaging (red) at ball bearing (BB) position midline with no pod block, where MV imaging showed comparable result with IDENTIFY overall. The scale of plots is shown in the left figure

Figure 4 shows the median and range of magnitude of translation residual error $|\varepsilon^{\mathrm{mid},\mathrm{pod}}\left(\theta \right)|$ at the BB position midline with no pod block (left), with left pod block (middle), and with right pod block (right). Figure 5 shows the difference in IDENTIFY compared to linac trajectory log and MV imaging for all five couch rotation angles at the BB position midline with no pod block.

## 4 DISCUSSION

Previous studies have utilized calculating the accuracy of SI systems by setting a phantom at either static couch positions or with regular, that is, sinusoidal, breathing patterns and comparing the difference between the SI systems and the input couch positions.^19^, ^20^, ^21^ The method presented in this paper uses automated couch motion in developer mode that increases the efficiency of measurements and enables the measurement of a large number of data points. The use of MV imaging also accounts for the inherent offset of the phantom as the couch is rotated due to couch walkout. Since a portion of the SI reported offset is due to this walkout, this method enables SI system users to characterize the residual errors of their system that are displayed in excess of couch walkout. The use of MV imaging and linac trajectory logs also provides a more accurate recording of couch position rather than using user input couch locations.

For the selection of MV images over linac trajectory log for longitudinal and lateral directions, MV imaging (red) showed comparable agreement with IDENTIFY compared to linac trajectory log (green) overall based on Figure 5. From Figure 2, we noticed that MV images were comparable to the linac trajectory log when compared to IDENTIFY. Moreover, it is not possible to subtract couch walkout from SI reported offset from linac trajectory log. From our experience, IDENTIFY displayed a larger error when there was a sudden movement of the couch rather than a smooth slow transition.

There was a difference between BB position placements based on Table 2. The absolute range of residual error difference between the BB position anterior or posterior to the BB position midline with no pod block was [0.01, 0.40] mm. The mean difference in the median residual error when the BB position was moved from midline to anterior or posterior based on Table 2 was 0.15 mm.

There was also a difference between pods blocked and pods not blocked based on Table 3. With the pod blocked, an absolute range of [0.01, 0.37] mm of residual error difference of the pod blocked compared to the pod not blocked was seen based on Table 3. The range of the median pod blocked residual error was [0.09, 0.79] mm, which was larger than its no pod blocked counterpart of [0.11, 0.61] mm. There were anecdotal reports that gantry motion could increase the SI reported offsets due to pod blocking.^22^ Utilizing the method in this paper, we were able to systematically test the SI residual error at various couch angles while blocking the pod that would typically be blocked during treatment by gantry motion. In Figure 3, a larger residual error magnitude and range are shown for both left and right pods blocked as well. The average residual error difference was 0.10 mm for pod block to no pod block at all BB positions.

The purpose of the study is to present a methodology of using automated six degree‐of‐freedom couch movement to test the uncertainty of the SI system in the reader's clinics. With the results from this study, clinics can choose their thresholds for stopping treatment based on their SI system's residual error in conjunction with the desired thresholds for patient motion. Because of the SI system residual error, we recommend radiographic imaging for patient repositioning. For example, with the results shown, we believe that an SI threshold of 0.80 mm could be set to stop treatment for intracranial SRS when there is no pod blocked for our system. This allows for 0.30 mm of intrinsic IDENTIFY uncertainty and patient movement of 0.50 mm; therefore, SI reported offset exceeding 0.80 mm would require reconfirming patient position with radiographic imaging. However, if the target is located more anteriorly or posteriorly in reference to the skull or if there is a pod blocked, we would increase this threshold to 0.95 or 0.90 mm, which comes from the increase in intrinsic uncertainty of IDENTIFY when the target position is not centered on the midline in the skull or when no pod is blocked. Using this method, SI users could determine their own threshold for repeating radiographic imaging.

## 5 CONCLUSION

This technique demonstrated a robust method for testing the uncertainty of a SI system. Automated six degree‐of‐freedom couch motion can be used to test the residual error of a SI system by incorporating MV images and linac trajectory log analysis. These data can be used to set the thresholds for decision‐making during SI tracking in SRS treatment.

## CONFLICT OF INTEREST

The study was supported by Varian Medical Systems.

## AUTHOR CONTRIBUTIONS

All the authors made substantial contributions to the conception and design of the work; the acquisition, analysis, and interpretation of data for the work; drafting the work and revising it critically for important intellectual content; gave final approval of the version to be published; and agrees to be accountable for all aspects of the work in ensuring that questions related to the accuracy or integrity of any part of the work are appropriately investigated and resolved.

## Supporting information

Supporting Information

Supporting Information
