# Supplementary material for: Analysis of a surface imaging system using a six degree‐of‐freedom couch
Source: J Appl Clin Med Phys. 2022 Jul 12;23(8):e13697. doi: 10.1002/acm2.13697 (PMC9359042; doi:10.1002/acm2.13697)
Supplement: Supplementary file 1 — Supporting Information [file ACM2-23-e13697-s002.docx]

**Supplementary material 1**

**Table S-1.** Summary of median and range of translational error, mean and standard deviation of roll, pitch and yaw at BB position anterior, midline and posterior with no pod block, left pod block and right pod block.

| BB Anterior | | | | | | | | | | |
| --- | --- | --- | --- | --- | --- | --- | --- | --- | --- | --- |
| no pod block | Table Angle θ(°) | median $\left\vert\varepsilon^{BB,pod}\left( \theta\right) \right\vert$ (mm) | min $\left\vert\varepsilon^{BB,pod}\left( \theta\right) \right\vert$ (mm) | max $\left\vert\varepsilon^{BB,pod}\left( \theta\right) \right\vert$ (mm) | mean $\varepsilon_{Roll}^{BB, pod}\left( \theta\right)$ (°) | std $\varepsilon_{Roll}^{BB, pod}\left( \theta\right)$ (°) | mean $\varepsilon_{Pitch}^{BB, pod}\left( \theta\right)$ (°) | std $\varepsilon_{Pitch}^{BB, pod}\left( \theta\right)$ (°) | mean $\varepsilon_{Yaw}^{BB, pod}\left( \theta\right)$ (°) | std $\varepsilon_{Yaw}^{BB, pod}\left( \theta\right)$ (°) |
|  | 0 | 0.14 | 0.06 | 0.24 | 0.00 | 0.02 | -0.03 | 0.01 | 0.00 | 0.02 |
|  | 45 | 0.23 | 0.18 | 0.34 | -0.10 | 0.01 | -0.19 | 0.01 | 0.08 | 0.02 |
|  | 90 | 0.40 | 0.26 | 0.46 | 0.04 | 0.02 | -0.43 | 0.02 | -0.02 | 0.03 |
|  | 270 | 0.35 | 0.21 | 0.48 | 0.29 | 0.02 | -0.16 | 0.02 | -0.11 | 0.03 |
|  | 315 | 0.37 | 0.25 | 0.56 | 0.17 | 0.02 | 0.08 | 0.01 | -0.05 | 0.02 |
| left pod block | 0 | 0.25 | 0.12 | 0.33 | 0.03 | 0.01 | -0.05 | 0.02 | -0.04 | 0.02 |
|  | 45 | 0.42 | 0.29 | 0.50 | -0.08 | 0.02 | -0.15 | 0.01 | 0.07 | 0.02 |
|  | 90 | 0.63 | 0.38 | 0.76 | -0.05 | 0.02 | -0.41 | 0.04 | 0.05 | 0.03 |
| right pod block | 0 | 0.13 | 0.05 | 0.27 | -0.06 | 0.02 | -0.07 | 0.02 | 0.02 | 0.03 |
|  | 270 | 0.33 | 0.28 | 0.37 | 0.43 | 0.03 | -0.10 | 0.02 | -0.09 | 0.04 |
|  | 315 | 0.50 | 0.29 | 0.64 | 0.19 | 0.02 | 0.10 | 0.02 | -0.09 | 0.01 |
| BB Midline | | | | | | | | | | |
| no pod block | 0 | 0.13 | 0.07 | 0.21 | 0.00 | 0.01 | 0.01 | 0.01 | -0.01 | 0.01 |
|  | 45 | 0.16 | 0.11 | 0.26 | -0.12 | 0.01 | -0.16 | 0.01 | 0.05 | 0.01 |
|  | 90 | 0.61 | 0.50 | 0.68 | 0.03 | 0.02 | -0.44 | 0.02 | 0.06 | 0.03 |
|  | 270 | 0.49 | 0.42 | 0.55 | 0.34 | 0.01 | -0.05 | 0.01 | -0.03 | 0.02 |
|  | 315 | 0.55 | 0.38 | 0.72 | 0.18 | 0.01 | 0.09 | 0.01 | -0.02 | 0.02 |
| left pod block | 0 | 0.14 | 0.10 | 0.24 | 0.03 | 0.02 | -0.03 | 0.03 | -0.03 | 0.02 |
|  | 45 | 0.19 | 0.09 | 0.25 | -0.11 | 0.02 | -0.19 | 0.02 | -0.01 | 0.01 |
|  | 90 | 0.64 | 0.51 | 0.75 | 0.02 | 0.03 | -0.39 | 0.04 | 0.09 | 0.04 |
| right pod block | 0 | 0.09 | 0.05 | 0.19 | -0.02 | 0.03 | -0.03 | 0.03 | 0.01 | 0.03 |
|  | 270 | 0.48 | 0.40 | 0.52 | 0.43 | 0.03 | -0.06 | 0.03 | -0.06 | 0.05 |
|  | 315 | 0.70 | 0.65 | 0.78 | 0.20 | 0.02 | 0.09 | 0.01 | 0.00 | 0.01 |
| BB Posterior | | | | | | | | | | |
| no pod block | 0 | 0.11 | 0.06 | 0.23 | -0.02 | 0.02 | 0.01 | 0.01 | -0.01 | 0.01 |
|  | 45 | 0.19 | 0.10 | 0.43 | -0.16 | 0.02 | -0.14 | 0.01 | 0.05 | 0.02 |
|  | 90 | 0.20 | 0.12 | 0.33 | -0.04 | 0.03 | -0.41 | 0.01 | 0.03 | 0.02 |
|  | 270 | 0.21 | 0.12 | 0.37 | 0.32 | 0.02 | -0.04 | 0.02 | 0.00 | 0.02 |
|  | 315 | 0.41 | 0.31 | 0.56 | 0.14 | 0.01 | 0.10 | 0.01 | 0.02 | 0.02 |
| left pod block | 0 | 0.23 | 0.15 | 0.30 | -0.02 | 0.02 | -0.05 | 0.03 | -0.05 | 0.03 |
|  | 45 | 0.32 | 0.20 | 0.50 | -0.16 | 0.02 | -0.13 | 0.02 | 0.05 | 0.02 |
|  | 90 | 0.38 | 0.28 | 0.60 | -0.05 | 0.03 | -0.38 | 0.03 | 0.01 | 0.02 |
| right pod block | 0 | 0.24 | 0.07 | 0.33 | -0.08 | 0.02 | 0.00 | 0.04 | 0.02 | 0.03 |
|  | 270 | 0.23 | 0.12 | 0.40 | 0.44 | 0.03 | -0.09 | 0.03 | -0.03 | 0.03 |
|  | 315 | 0.79 | 0.67 | 0.96 | 0.17 | 0.03 | 0.16 | 0.01 | 0.04 | 0.02 |
