# Supplementary material for: Analysis of a surface imaging system using a six degree‐of‐freedom couch
Source: J Appl Clin Med Phys. 2022 Jul 12;23(8):e13697. doi: 10.1002/acm2.13697 (PMC9359042; doi:10.1002/acm2.13697)
Supplement: Supplementary file 2 — Supporting Information [file ACM2-23-e13697-s001.docx]

**Supplementary material 2**

**.xml** The following .xml file is an example at couch 90° used in developer mode on Varian Edge machine for automatic couch movement to deliver 550MU at 11 couch positions with the 1^st^ couch position at isocenter.

<?xml version="1.0" encoding="UTF-8"?>

-<VarianResearchBeam SchemaVersion="1.0">

-<SetBeam>

<Id>1</Id>

<MLCModel>NDS120HD</MLCModel>

-<TolTable>

<CouchVrt>0.005</CouchVrt>

<CouchLat>0.005</CouchLat>

<CouchLng>0.005</CouchLng>

<CouchRtn>0.05</CouchRtn>

<CouchPit>0.05</CouchPit>

<CouchRol>0.05</CouchRol>

</TolTable>

-<VelTable>

<CouchLat>1</CouchLat>

<CouchLng>1</CouchLng>

</VelTable>

<Accs/>

-<ControlPoints>

-<Cp>

-<SubBeam>

<Seq>0</Seq>

<Name>Beam ON</Name>

</SubBeam>

<Energy>6x</Energy>

<Mu>0</Mu>

<DRate>600</DRate>

<GantryRtn>180</GantryRtn>

<CollRtn>180</CollRtn>

<CouchVrt>107.76</CouchVrt>

<CouchLat>99.61</CouchLat>

<CouchLng>49.1</CouchLng>

<CouchRtn>90</CouchRtn>

<CouchPit>0</CouchPit>

<CouchRol>0</CouchRol>

<Y1>5</Y1>

<Y2>5</Y2>

<X1>5</X1>

<X2>5</X2>

</Cp>

-<Cp>

<Mu>0</Mu>

</Cp>

-<Cp>

<Mu>0</Mu>

</Cp>

-<Cp>

<Mu>50</Mu>

</Cp>

-<Cp>

<Mu>50</Mu>

<CouchVrt>107.1334</CouchVrt>

<CouchLat>99.6009</CouchLat>

<CouchLng>49.0606</CouchLng>

<CouchRtn>89.9053</CouchRtn>

<CouchPit>-0.23319</CouchPit>

<CouchRol>0.11356</CouchRol>

</Cp>

-<Cp>

<Mu>50</Mu>

</Cp>

-<Cp>

<Mu>50</Mu>

</Cp>

-<Cp>

<Mu>100</Mu>

</Cp>

-<Cp>

<Mu>100</Mu>

<CouchVrt>107.2517</CouchVrt>

<CouchLat>99.8983</CouchLat>

<CouchLng>49.022</CouchLng>

<CouchRtn>89.8393</CouchRtn>

<CouchPit>-0.17467</CouchPit>

<CouchRol>-0.63667</CouchRol>

</Cp>

-<Cp>

<Mu>100</Mu>

</Cp>

-<Cp>

<Mu>100</Mu>

</Cp>

-<Cp>

<Mu>150</Mu>

</Cp>

-<Cp>

<Mu>150</Mu>

<CouchVrt>107.8302</CouchVrt>

<CouchLat>99.8223</CouchLat>

<CouchLng>49.1023</CouchLng>

<CouchRtn>90.5834</CouchRtn>

<CouchPit>0.05558</CouchPit>

<CouchRol>-0.29256</CouchRol>

</Cp>

-<Cp>

<Mu>150</Mu>

</Cp>

-<Cp>

<Mu>150</Mu>

</Cp>

-<Cp>

<Mu>200</Mu>

</Cp>

-<Cp>

<Mu>200</Mu>

<CouchVrt>107.9331</CouchVrt>

<CouchLat>99.5979</CouchLat>

<CouchLng>49.1158</CouchLng>

<CouchRtn>90.0655</CouchRtn>

<CouchPit>0.019039</CouchPit>

<CouchRol>-0.14761</CouchRol>

</Cp>

-<Cp>

<Mu>200</Mu>

</Cp>

-<Cp>

<Mu>200</Mu>

</Cp>

-<Cp>

<Mu>250</Mu>

</Cp>

-<Cp>

<Mu>250</Mu>

<CouchVrt>107.868</CouchVrt>

<CouchLat>99.6835</CouchLat>

<CouchLng>49.0189</CouchLng>

<CouchRtn>89.935</CouchRtn>

<CouchPit>0.027282</CouchPit>

<CouchRol>-0.095865</CouchRol>

</Cp>

-<Cp>

<Mu>250</Mu>

</Cp>

-<Cp>

<Mu>250</Mu>

</Cp>

-<Cp>

<Mu>300</Mu>

</Cp>

-<Cp>

<Mu>300</Mu>

<CouchVrt>106.5471</CouchVrt>

<CouchLat>99.5085</CouchLat>

<CouchLng>48.9869</CouchLng>

<CouchRtn>89.9745</CouchRtn>

<CouchPit>-0.37932</CouchPit>

<CouchRol>0.32232</CouchRol>

</Cp>

-<Cp>

<Mu>300</Mu>

</Cp>

-<Cp>

<Mu>300</Mu>

</Cp>

-<Cp>

<Mu>350</Mu>

</Cp>

-<Cp>

<Mu>350</Mu>

<CouchVrt>105.2459</CouchVrt>

<CouchLat>99.3557</CouchLat>

<CouchLng>48.7925</CouchLng>

<CouchRtn>89.7009</CouchRtn>

<CouchPit>-0.8337</CouchPit>

<CouchRol>0.3718</CouchRol>

</Cp>

-<Cp>

<Mu>350</Mu>

</Cp>

-<Cp>

<Mu>350</Mu>

</Cp>

-<Cp>

<Mu>400</Mu>

</Cp>

-<Cp>

<Mu>400</Mu>

<CouchVrt>107.1985</CouchVrt>

<CouchLat>99.7161</CouchLat>

<CouchLng>48.9852</CouchLng>

<CouchRtn>90.2521</CouchRtn>

<CouchPit>-0.19237</CouchPit>

<CouchRol>-0.12409</CouchRol>

</Cp>

-<Cp>

<Mu>400</Mu>

</Cp>

-<Cp>

<Mu>400</Mu>

</Cp>

-<Cp>

<Mu>450</Mu>

</Cp>

-<Cp>

<Mu>450</Mu>

<CouchVrt>107.659</CouchVrt>

<CouchLat>99.3835</CouchLat>

<CouchLng>49.0334</CouchLng>

<CouchRtn>90.246</CouchRtn>

<CouchPit>-0.063163</CouchPit>

<CouchRol>0.52714</CouchRol>

</Cp>

-<Cp>

<Mu>450</Mu>

</Cp>

-<Cp>

<Mu>450</Mu>

</Cp>

-<Cp>

<Mu>500</Mu>

</Cp>

-<Cp>

<Mu>500</Mu>

<CouchVrt>107.7917</CouchVrt>

<CouchLat>99.7126</CouchLat>

<CouchLng>49.0986</CouchLng>

<CouchRtn>90.0816</CouchRtn>

<CouchPit>0.0022552</CouchPit>

<CouchRol>-0.20839</CouchRol>

</Cp>

-<Cp>

<Mu>500</Mu>

</Cp>

-<Cp>

<Mu>500</Mu>

</Cp>

-<Cp>

<Mu>550</Mu>

</Cp>

</ControlPoints>

-<ImagingParameters>

<DuringTreatment/>

-<ImagingPoints>

-<ImagingPoint>

<Cp>0</Cp>

-<Mvd>

-<Positions>

<Lat>0</Lat>

<Lng>0</Lng>

<Vrt>-70</Vrt>

<Pitch>0</Pitch>

</Positions>

</Mvd>

</ImagingPoint>

-<ImagingPoint>

<Cp>2.5</Cp>

-<Acquisition>

<AcquisitionId>1</AcquisitionId>

-<AcquisitionSpecs>

<Handshake>true</Handshake>

</AcquisitionSpecs>

-<AcquisitionParameters>

<ImageMode id="Highres"/>

<CalibrationSet>DefaultCalibrationSetId</CalibrationSet>

<MV/>

</AcquisitionParameters>

</Acquisition>

</ImagingPoint>

-<ImagingPoint>

<Cp>6.5</Cp>

-<Acquisition>

<AcquisitionId>2</AcquisitionId>

-<AcquisitionSpecs>

<Handshake>true</Handshake>

</AcquisitionSpecs>

-<AcquisitionParameters>

<ImageMode id="Highres"/>

<CalibrationSet>DefaultCalibrationSetId</CalibrationSet>

<MV/>

</AcquisitionParameters>

</Acquisition>

</ImagingPoint>

-<ImagingPoint>

<Cp>10.5</Cp>

-<Acquisition>

<AcquisitionId>3</AcquisitionId>

-<AcquisitionSpecs>

<Handshake>true</Handshake>

</AcquisitionSpecs>

-<AcquisitionParameters>

<ImageMode id="Highres"/>

<CalibrationSet>DefaultCalibrationSetId</CalibrationSet>

<MV/>

</AcquisitionParameters>

</Acquisition>

</ImagingPoint>

-<ImagingPoint>

<Cp>14.5</Cp>

-<Acquisition>

<AcquisitionId>4</AcquisitionId>

-<AcquisitionSpecs>

<Handshake>true</Handshake>

</AcquisitionSpecs>

-<AcquisitionParameters>

<ImageMode id="Highres"/>

<CalibrationSet>DefaultCalibrationSetId</CalibrationSet>

<MV/>

</AcquisitionParameters>

</Acquisition>

</ImagingPoint>

-<ImagingPoint>

<Cp>18.5</Cp>

-<Acquisition>

<AcquisitionId>5</AcquisitionId>

-<AcquisitionSpecs>

<Handshake>true</Handshake>

</AcquisitionSpecs>

-<AcquisitionParameters>

<ImageMode id="Highres"/>

<CalibrationSet>DefaultCalibrationSetId</CalibrationSet>

<MV/>

</AcquisitionParameters>

</Acquisition>

</ImagingPoint>

-<ImagingPoint>

<Cp>22.5</Cp>

-<Acquisition>

<AcquisitionId>6</AcquisitionId>

-<AcquisitionSpecs>

<Handshake>true</Handshake>

</AcquisitionSpecs>

-<AcquisitionParameters>

<ImageMode id="Highres"/>

<CalibrationSet>DefaultCalibrationSetId</CalibrationSet>

<MV/>

</AcquisitionParameters>

</Acquisition>

</ImagingPoint>

-<ImagingPoint>

<Cp>26.5</Cp>

-<Acquisition>

<AcquisitionId>7</AcquisitionId>

-<AcquisitionSpecs>

<Handshake>true</Handshake>

</AcquisitionSpecs>

-<AcquisitionParameters>

<ImageMode id="Highres"/>

<CalibrationSet>DefaultCalibrationSetId</CalibrationSet>

<MV/>

</AcquisitionParameters>

</Acquisition>

</ImagingPoint>

-<ImagingPoint>

<Cp>30.5</Cp>

-<Acquisition>

<AcquisitionId>8</AcquisitionId>

-<AcquisitionSpecs>

<Handshake>true</Handshake>

</AcquisitionSpecs>

-<AcquisitionParameters>

<ImageMode id="Highres"/>

<CalibrationSet>DefaultCalibrationSetId</CalibrationSet>

<MV/>

</AcquisitionParameters>

</Acquisition>

</ImagingPoint>

-<ImagingPoint>

<Cp>34.5</Cp>

-<Acquisition>

<AcquisitionId>9</AcquisitionId>

-<AcquisitionSpecs>

<Handshake>true</Handshake>

</AcquisitionSpecs>

-<AcquisitionParameters>

<ImageMode id="Highres"/>

<CalibrationSet>DefaultCalibrationSetId</CalibrationSet>

<MV/>

</AcquisitionParameters>

</Acquisition>

</ImagingPoint>

-<ImagingPoint>

<Cp>38.5</Cp>

-<Acquisition>

<AcquisitionId>10</AcquisitionId>

-<AcquisitionSpecs>

<Handshake>true</Handshake>

</AcquisitionSpecs>

-<AcquisitionParameters>

<ImageMode id="Highres"/>

<CalibrationSet>DefaultCalibrationSetId</CalibrationSet>

<MV/>

</AcquisitionParameters>

</Acquisition>

</ImagingPoint>

-<ImagingPoint>

<Cp>42.5</Cp>

-<Acquisition>

<AcquisitionId>11</AcquisitionId>

-<AcquisitionSpecs>

<Handshake>true</Handshake>

</AcquisitionSpecs>

+<AcquisitionParameters>

</Acquisition>

</ImagingPoint>

</ImagingPoints>

<ImagingTolerances/>

</ImagingParameters>

</SetBeam>

</VarianResearchBeam>
